# Anti-inflammatory Effect of Amitriptyline on Ulcerative Colitis in Normal and Reserpine-Induced Depressed Rats

**Published:** 2016

**Authors:** Ehsan Fattahian, Valiollah Hajhashemi, Mohammad Rabbani, Mohsen Minaiyan, Parvin Mahzouni

**Affiliations:** a *Department of Pharmacology and Isfahan Pharmaceutical Sciences Research Center, School of Pharmacy and Pharmaceutical Sciences, Isfahan University of Medical Sciences, Isfahan, Iran.*; b *Department of Clinical Pathology, School of Medicine, Isfahan University of Medical Sciences, Isfahan, Iran.*

**Keywords:** Ulcerative colitis, Acetic acid, Depressive disorders, Amitriptyline, Reserpine, Rats

## Abstract

Depressive disorders are more common among persons with chronic diseases such as inflammatory bowel disease and anti-inflammatory effect of some antidepressants such as amitriptyline has been reported. Acetic acid colitis was induced in both reserpinised (depressed) and non-reserpinised (normal) rats. Reserpinised groups received reserpine (6 mg/kg, i.p.) one hour prior to colitis induction. Then Amitriptyline (5, 10, 20 mg/kg, i.p.) was administered to separate groups of male Wistar rats. All treatments were carried out two hours after colitis induction and continued daily for four days. Dexamethasone (1 mg/kg) and normal saline (1 mL/kg) were used in reference and control groups, respectively. At day five, animals were euthanized and colonic tissue injuries were assessed macroscopically and pathologically. Myeloperoxidase activity as a marker of neutrophil infiltration was also measured in colonic tissues. Results showed that reserpine (6 mg/kg, i.p.) intensified colitic condition. Compared to control, amitriptyline (10, 20 mg/kg) and dexamethasone significantly decreased weight of colon and ulcer index in normal and reserpine-induced depressed rats. Myeloperoxidase activity and pathological assessments also proved anti-inflammatory effect of amitriptyline.

Our results suggest that amitriptyline, a tricyclic antidepressant, could reduce inflammatory and ulcerative injuries of colon both in normal and depressed rats. So among the wide spread anti-depressant drugs, amitriptyline is a good choice to treat depression comorbidities in patients with IBD.

## Introduction

 Ulcerative colitis (UC) and Crohn's disease are two idiopathic inflammatory bowel disorders (1). Although the etiology of inflammatory bowel disorders (IBD) remains unclear, there is evidence that it involves immune, genetic and environmental factors, which are, in turn, related to the initiation and progression of colitis (2, 3). The main clinical manifestations are abdominal pain, diarrhea, bloody mucous and purulent stools, recurrent attacks, and relapse. The main treatment of patients with IBD has been the use of anti-inflammatory and immunomodulatory drugs and sometimes antibiotics to relieve the symptoms (4). Although UC initially was considered to represent a psychosomatic disease, the role of behavioral factors in IBD is controversial (5). As our awareness of the role of the immune system in IBD has increased, less attention has been paid to the role of the nervous system and behavioral factors in the expression of these conditions, despite some florid demonstrations of a tangible modulatory role of the nervous system in precipitating relapse in humans (6). There are several possible explanations for the co-occurrence of depression or anxiety and IBD. These two psychiatric disorders might indeed predispose people to IBD or, conversely, IBD might predispose people to depression or anxiety (7). Clinical and experimental evidences indicate that intestinal inflammatory conditions can be exacerbated by behavioral conditions such as depression (8). Studies in patients with UC demonstrated that depression was a risk factor of relapses and that psychological distress induced systemic and mucosal pro-inflammatory responses (9, 10). In addition, depression is associated with alterations in the immune system. The “macrophage theory of depression” provides an explanation for the significant association of depression with diseases where macrophage activation occurs. Secretion of pro-inflammatory cytokines such as interleukin (IL)-1, tumor necrosis factor alfa (TNF-alfa) and interferon gamma (IFN-gamma) is associated with major depression (11). Depression has also been associated with hyper-activation of the immune system resulting in increased TH_1 _cytokines (12). There is growing evidence that anti-depressive treatment may influence the production of pro and anti-inflammatory cytokines. Anti-depressants may exert therapeutic effects via inhibition of pro-inflammatory cytokine production. The effects of antidepressants on the cytokines have also been investigated *in-vitro*

 in cell cultures (13). The first evidence indicating that antidepressants have anti-inflammatory effects appeared four decades ago. Martelli *et al*. showed that inhibition of catecholamine uptake by antidepressant drugs, initiates a number of reactions which ultimately lead to an inhibition of the inflammatory response (14). An *ex-vivo* study revealed that the selective serotonin reuptake inhibitors (SSRIs), paroxetine and sertraline, have a modulatory effect on some components of the human immune system. The phenotypic effect is characterized by the inhibition of T-cell proliferation and TNF-alfa secretion. These effects seem to be related to the suppressive effect of SSRIs on the expression of genes involved in cell proliferation (most consistent Cdc6). In addition, inflammatory protein (such as STAT3 and COX2) expression was suppressed, apparently by the SSRIs (12). Furthermore, experimental evidence is accumulating that various types of antidepressants exert anti-inflammatory effects by decreasing pro-inflammatory cytokine levels or increasing anti-inflammatory cytokine levels (15). Amitriptyline is a tricyclic antidepressant (TCA) acts as a dual serotonergic and noradrenergic reuptake inhibitor. It is widely used in the management of major depression and different types of pain, including both inflammatory (rheumatoid arthritis and fibromyalgia) and neuropathic pain (neuropathic pain and post herpetic neuralgia) (16-18). Additionally anti-inflammatory effect of amitriptyline on carrageenan-induced paw edema has been evaluated (19, 20). Therefore, the purpose of the present study was to evaluate the effect of amitriptyline on UC induced by acetic acid in normal and reserpine-induced depressed rats.

## Experimental

*Materials*

Amitriptyline hydrochloride powder was a gift from Daroupakhsh Pharmaceutical Company (Tehran, Iran). Dexamethasone was also a gift from Raha Pharmaceutical Company (Isfahan, Iran). Reserpine, hexadecyltrimethyl-ammonium bromide (HTAB) and O-dianisidinedihydrochloride were purchased from Sigma chemical company (St. Louis, USA). Formalin solution 35% w/w, glacial acetic acid and diethyl etheroxide were purchased from Merck (Darmstadt, Germany). All other solvents and chemicals were of analytical grade.

*Animals*

 Male Wistar rats (200 ± 20 g) from the animal house of the School of Pharmacy and Pharmaceutical Sciences of Isfahan University of Medical Sciences were used. The animals were kept in separate cages in controlled conditions (standard temperature, humidity and light/dark 12/12 cycle) on normal chow-pellet diet and free water access. Rats were fasted for 24 h before induction of colitis with free access to water. The animal study was approved by the guideline of the ethical committee of Isfahan University of Medical Sciences.

*Behavioral tests*

*Determination of reserpine dose for induction of depression*

Twenty four male Wistar rats were divided into four groups (n=6) of normal and reserpine (3, 6, 12 mg/kg, i.p.) treated rats. Sham group maintained on regular rat food and drinking water *ad libitum* and reserpine groups received one dose of reserpine in the beginning of experiment. After four days all animals were subjected to the forced swimming test.

*Evaluation of anti-depressant effect of amitriptyline in reserpine treated rats*

 Thirty six male Wistar rats were divided into six groups (n=6) as normal, control and test groups as following:

Sham group: received i.p. injection of normal saline daily for four days.

Control group: received reserpine (6 mg/kg, i.p.) at first day and daily injections of normal saline for four days.

Test groups: received reserpine (6 mg/kg, i.p.) at first day and daily amitriptyline (2.5, 5, 10, 20mg/kg, i.p.) for four days. 

 Then all animals were subjected to the forced swimming test.

*Forced swimming test in rats*

 The swimming test was performed on two consecutive days according to Porsolt *et al*. (21). 

On the first day (the third day of the experiment) the rats were individually placed in a cylinder containing water 15 cm in height at 25 °C for 15min. On the following day (4th day) the rats were again immersed in water and total duration of immobility was measured for 5 min. The immobility time was regarded as the time that rats spent floating in the water without struggling and making only those movements necessary to keep their heads above water.

*Induction of experimental colitis*

Colitis was induced according to the MacPherson and Pfeiffer’s method (22). Briefly, animals were deprived of food for 24 h before the induction of colitis but allowed free access to water. Rats were slightly anesthetized with ether and a medical grade polyurethane tube (diameter 2 mm) was inserted into the anus and the tip was advanced to 8 cm proximal to the anus verge. Two mL of acetic acid (4% v/v in 0.9% saline) or normal saline alone (control animals) was instilled into the colon through cannula for 30 s, after which fluid was withdrawn. Animals were allowed to hang in air by holding their tails for 40 second. This prevents spillage of solution from rectum.

*Administration of drugs*

The rats were assigned to Sham, control, test, and reference groups of six as following:

Sham groups: cannulation was accomplished without induction of colitis (normal saline was administered instead of acetic acid), and rats also received normal saline (2 mL/kg, i.p.). Control groups: treated with normal saline (2 mL/kg, i.p.), respectively after induction of colitis. Test groups: non-reserpine treated groups received amitriptyline (5, 10, 20 mg/kg, i.p.) and reserpine treated groups which received reserpine (6mg/kg, i.p.), 1 h prior to induction of colitis and then treated with amitriptyline (5, 10, 20 mg/kg, i.p.). Reference groups: treated with dexamethasone (1mg/kg, i.p.). All treatments were carried out 2 h after colitis induction and continued daily for 4 days.

*Assessment of colonic injury and inflammation*

Twenty four hours after administration of the last dose of amitriptyline, the rats were euthanized with a high dose of ether and a midline incision was made in the abdomen. The 8-cm distal segment of the colon was removed, opened longitudinally and slightly cleaned in physiological saline to remove fecal residues and processed for assessment by macroscopic, histological scores and biochemical markers (23).

*Macroscopic assessment*

For each specimen, distal colon wet weight (g) (8 cm from the anus) were measured. Then, tissue was fixed on a white plastic sheet and a photo was taken using an appropriately adjusted Nikon camera (Coolpix p100). Severity of gross macroscopic injury was assessed using a scoring system (24): 0= no macroscopic changes, 1= mucosal erythema only, 2= mild mucosal edema, slight bleeding, or slight erosion, 3= moderate edema, bleeding ulcers, or erosions, and 4= severe ulceration, erosions, edema, and tissue necrosis. Ulcer area was assessed by Fiji-win 32 software, an image processing and analysis software inspired by NIH Image for the Macintosh (25). For each specimen ulcer index was calculated using the following equation:

Ulcer Index= Ulcer area (Cm^2^) + Macroscopic score (26).

*Histopathological studies*

Sections of colon specimens were fixed in phosphate buffered formalin solution (10%), embedded in paraffin, sliced into 5 μm-thick sections, then stained with haematoxylin and eosin (H & E) and evaluated by light microscopy for morphological changes (27). Inflammation extent and severity and crypt damage were considered to assess the colonic damage from the histopathological point of view. A scale was defined for each of the four criteria based on the percent of damage which is presented in Table 1 (28). Total colitis was also calculated by summation the scores of inflammation severity, inflammation extent and crypt damage (29).

**Table 1:** Histological grading of colitis

**Scoring parameter**	**Score definition**
Inflammation severity	0 (None)
	1 (Mild)
	2 (Moderate)
	3 (Severe)
Inflammation extent	0 (None)
	1 (Mucosa)
	2 (Mucosa and submucosa)
	3 (Transmural)
	0 (None)
Crypt damage	1 (Basal 1/3 damaged)
	2 (Basal 2/3 damaged)
	3 (Crypts lost, surface epithelium present)
	4 (Crypts lost, surface epithelium lost)

*Determination of Colonic Myeloperoxidase (MPO) Activity*

 MPO activity was measured according to the modified method of Bradley *et al*. (30). In brief, colonic tissues were weighted and homogenized in 1 mL solution containing 0.5% (w/v) HTAB dissolved in 50 mm potassium phosphate bufferin, pH 6.0, in an ice bath using polytron homogenizer (50 mg tissue/mL). More buffers were added to obtain a concentration equivalent to 5 mL per 0.1 g of colon tissue and homogenized (15,000 rpm) for 4×45 s at 1 min intervals. then, the homogenate was sonicated in an ice bath for 10 s, then subjected to a sequence of freezing and thawing 3 times, and sonicated again for 10 s. then the homogenates were centrifuged for 15 min at 15,000 rpm at 4 °C. Then the supernatant decanted for analysis. The MPO activity in supernatants was assessed spectrophotometrically: 0.1 mL of the supernatant was added to 2.9 mL of 50 mM K3PO4 buffer (pH=6.0) containing O-dianisidinedihydrochloride (0.167 mg/mL) and 0.005% hydrogen peroxide. The absorbance of the reaction mixture was measured at 460 nm using a UV–Vis spectrophotometer. MPO activity was expressed in units (U) per gram tissue weight of wet tissue.

*Statistical analysis*

Parametric data are expressed as the mean ± S.E.M and non-parametric data are expressed as the median (range). One-way analysis of variance (ANOVA) followed by Tukey multiple comparisons were used to analyze the parametric data, using GraghPad Prism software (ver. 5.04). Non-parametric data were analyzed using Kruskal–Wallis followed by Mann-Whitney U test using SPSS ver.16. Differences were considered significant at P<0.05.

## Results

*Behavioral tests*

*Evaluation of the depressive reserpine dose*

**Figure 1:**
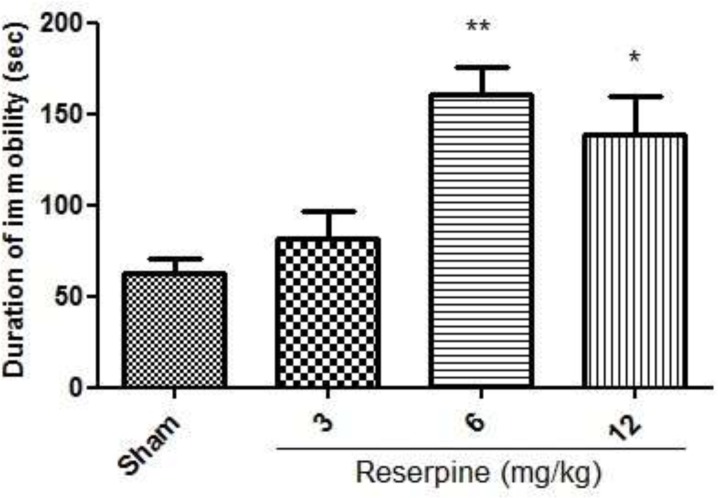
Effect of different doses of reserpine (3, 6, 12 mg/kg, i.p) in the rat forced swimming test; i.p. =intraperitoneally; Values are presented as mean ± S.E.M of six rats in each group; * P<0.05, ** P<0.01 compared to Sham, one-way ANOVA followed by Tukey test

As illustrated in Figure 1, i.p. injection of reserpine at doses of 6 mg/kg (p < 0.01), 12 mg/kg (p < 0.05) increased the time spent immobile during the forced swimming test as compared to the normal group. 

*Anti-depressant effect of amitriptyline on forced swimming test*

**Figure 2:**
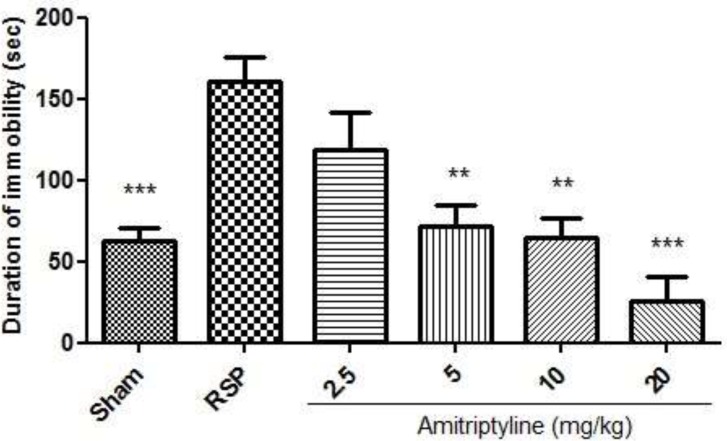
Effect of amitriptyline (2.5, 5, 10, 20 mg/kg, i.p.) on immobilization time (seconds) during forced swimming test in reserpinised (6 mg/kg) rats; i.p. =intraperitoneally, RSP= reserpine; Values are presented as mean ± S.E.M of six rats in each group; ** P< 0.01 and *** P< 0.001, compared to RSP, one-way ANOVA followed by Tukey test

 As it is shown in Figure 2, i.p. injection of amitriptyline at doses of 5, 10, 20 mg/kg significantly reduced the time spent immobile during the forced swimming test as compared to RSP (reserpine, 6 mg/kg) group.

*Macroscopic assessment of the colon*

 Twenty four hours after the instillation of 4 % acetic acid into the colon, the animals developed bloody diarrhea, weakness and decreased food intake. Macroscopic studies showed signs of damage, for example the presence of mucosal hyperemia and hemorrhagic lesions. Parameters including colon wet weight and ulcer index were used to evaluate the macroscopic damage of the colon.

*Weight of colon*

**Figure 3:**
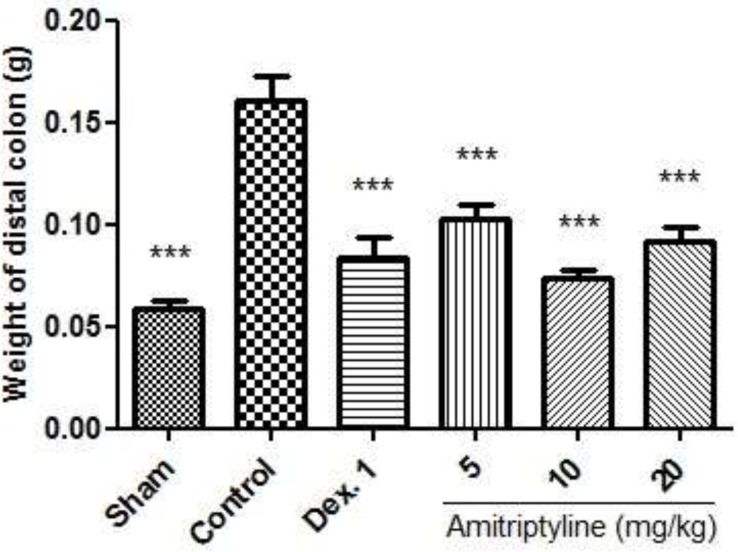
Effect of amitriptyline (5, 10, 20 mg/kg, i.p.) on weight of distal colon; i.p. = intraperitoneally, Dex. 1=dexamethasone (1 mg/kg); Values are presented as mean ± S.E.M of six rats in each group; *** P< 0.001 compared to control, one-way ANOVA followed by Tukey test

 As shown in Figure 3, no changes were observed in Sham group which suggests that handling and surgical procedure had no interference with experimental results. Treatment with dexamethasone as reference drug reduced the weight of 8 cm of wet colon compared to control (p<0.001). Amitriptyline at doses of 5, 10, 20 mg/kg, i.p. significantly reduced the weight of 8 cm of colon compared to control group after the induction of colitis (P<0.001).

**Figure 4.**
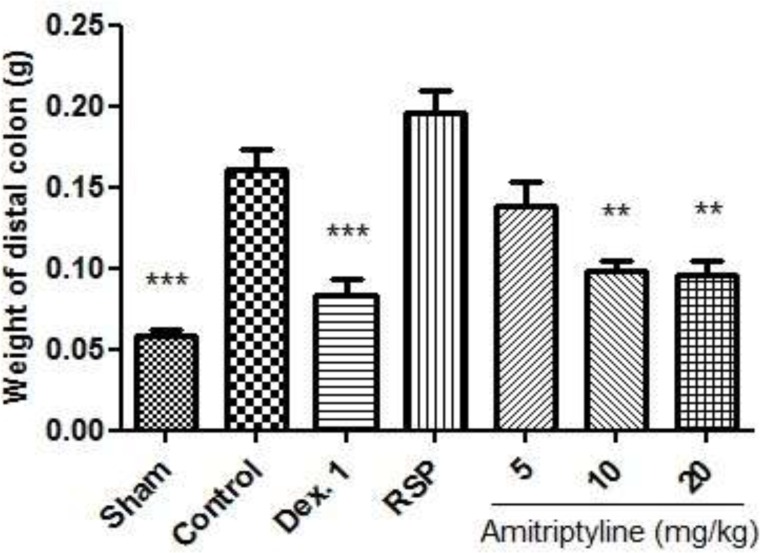
Effect of amitriptyline (5, 10, 20 mg/kg, i.p.) on weight of distal colon in reserpine (6mg/kg) induced depressed rats; i.p. =intraperitoneally, RSP= reserpine (6mg/kg), Dex.1 = dexamethasone (1mg/kg); Animals were also induced colitis; Values are presented as mean ± S.E.M of six rats in each group; ** P<0.01, *** P < 0.001 compared to control, one-way ANOVA followed by Tukey test

 In reserpine treated depressed rats which colitis was also induced, the severity of ulcers was enhanced and the average score was increased accordingly. Also injection of amitriptyline at doses of 10, 20 mg/kg, i.p. (P<0.01), and dexamethasone (P<0.001), significantly reduced the weight of colon compared with control group (Figure 4). 

*Evaluation of the ulcer index*

**Figure 5:**
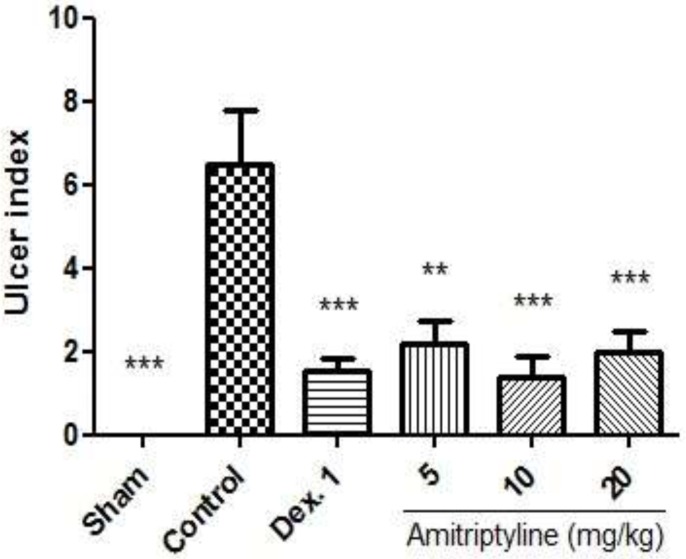
Effect of amitriptyline (5, 10, 20 mg/kg, i.p.) on ulcer index; i.p. =intraperitoneally, Dex. 1=dexamethasone (1mg/kg); Values are presented as mean ± S.E.M of six rats in each group; ** P < 0.01, *** P< 0.001 compared to control, one-way ANOVA followed by Tukey test

**Figure 6:**
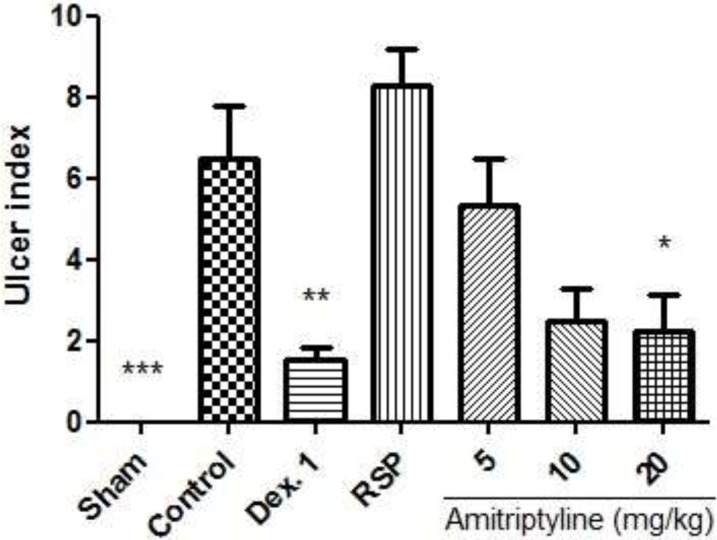
Effect of amitriptyline (5, 10, 20 mg/kg, i.p.) on ulcer index in reserpine induced (6mg/kg, i.p.) depressed rats; i.p. =intraperitoneally, RSP= reserpine (6mg/kg), Dex. 1 = dexamethasone (1mg/kg); Animals were also induced colitis; Values are presented as mean ± S.E.M of six rats in each group; * P<0.05, ** P<0.01 and *** P<0.001 compared to control, one-way ANOVA followed by Tukey test

 Severe inflammation, hemorrhage, ulcer, necrosis and thickened colon wall were found five days following induction of colitis in control group and RSP group, whereas macroscopic features of colon was quite intact in Sham group (Figures. 5, 6). As shown in Figure 5, amitriptyline at doses of 10, 20 mg/kg and dexamethasone (1 mg/kg) decreased ulcer index as compared to control group significantly (P<0.001). Also amitriptyline at doses of 5 mg/kg decreased ulcer index compared to control group significantly (P<0.01).

 In reserpine treated depressed rats which were also induced colitis, ulcer severity and ulcer index were exacerbated in RSP (reserpine 6 mg/kg) group. As illustrated in Figure 6, amitriptyline at dose of 20 mg/kg (P<0.05) and dexamethasone (P<0.01), significantly decreased ulcer index as compared to control group.

*Pathological assessment of the colon*

 Inflammation severity, inflammation extent and crypt damage were three parameters for scoring histological damage. The mean values of the histological scores of colitis in different groups of normal and reserpine induced depressed animals are presented in Tables 2 and 3.

**Table 2.** Effects of amitriptyline (AMT, 5, 10, 20 mg/kg, i.p.) on pathologic parameters of colitis induced by acetic acid in normal rats

**Group**	**Inflammation** **Severity (0-3)**	**Inflammation** **Extent (0-3)**	**Crypt Damage (0-4)**	**Total Colitis** **Index (0-10)**
**Sham**	0 (0-0)**	0 (0-0)**	0 (0-0)**	0 (0-0)**
**Control**	3 (2-3)	3 (2-3)	4 (1-4)	9.5 (6-10)
**Dex. 1**	0.5(0-1)**	0 (0-2)**	0 (0-2)**	1 (0-5)**
**AMT 5**	2 (0-3)	2 (0-3)	2 (0-4)	6.5 (0-9)
**AMT 10**	0.5 (0-1)**	0.5 (0-2)**	0 (0-1)**	1 (0-4)**
**AMT 20**	1 (0-1)**	1.5 (0-2)*	0 (0-0)**	2.5 (0-3)**

Dex.1=dexamethasone (1mg/kg), i.p.=intraperitoneally; Values are presented as median (range) of six rats in each group;

* P < 0.05,

** P< 0.01 compared to control, Mann-Whitney U test.

**Figure 7:**
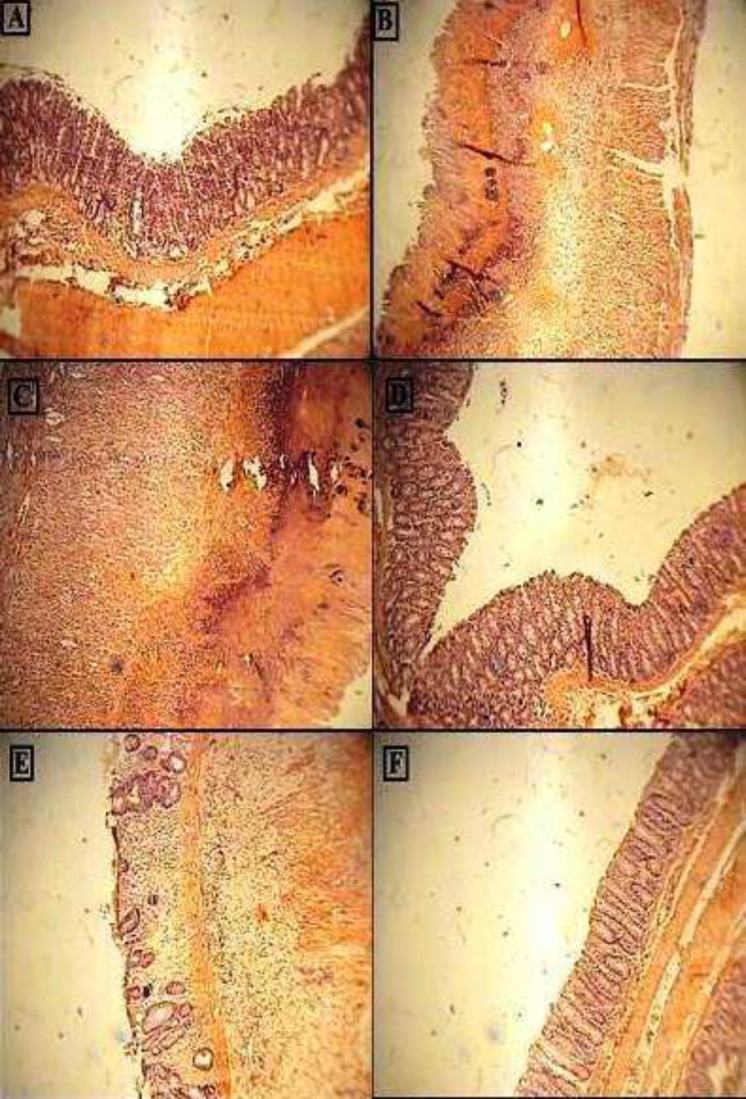
Photomicrographs of H&E stained paraffin sections of rat colonic tissues; (A) Normal intact mucosa from normal control animals showed intact epithelial surface. (B) Colitis induced by acetic acid in control group; Crypt damage, mucosal layers destruction and leukocyte infiltration are evident; (C) acetic acid-induced colitis in reserpine induced (6 mg/kg, i.p.) depressed rat showing massive necrotic destruction of epithelium; D, E and FColitis tissue treated with dexamethasone (1 mg/kg, i.p.), amitriptyline (10 mg/kg, i.p.), amitriptyline (20 mg/kg, i.p.) respectively, showing attenuated the extent and severity of the histological signs of cell damage;i.p. =intraperitoneally; Original Magnification*×*10

In Sham group, no pathological and histological damage was seen while control group and rats with reserpine (6 mg/kg) expressed necrotic destruction of epithelium hemorrhage, edema, inflammatory cellular infiltration, crypt damage, and ulceration at mucus and sub-mucosal layers (Table 2, Figure 7). Compared to control group, the histopathological changes in amitriptyline (10 mg/kg) or dexamethasone-treated groups, were significantly attenuated (p<0.01), as judged by re-epithelization of the mucosal layer, a decline in edema and reduced inflammatory cell recruitment in lamina propria. In this regard, no significant difference was seen between amitriptyline (10 mg/kg) and dexamethasone. Treatment with amitriptyline at dose of 20mg/kg also showed significant reduction in inflammation severity (p<0.01), inflammation extent (p<0.05) and crypt damage (p<0.01) compare to control group, while there was no significant differences between control and amitriptyline at dose of 5 mg/kg.

**Table 3.** Effects of amitriptyline (AMT, 5, 10, 20 mg/kg, i.p.) on pathologic parameters of colitis induced by acetic acid in reserpine induced (6 mg/kg, i.p.) depressed rats

**Group**	**Inflammation** **Severity (0-3)**	**Inflammation** **Extent (0-3)**	**Crypt Damage** **(0-4)**	**Total Colitis Index (0-10)**
**Sham**	0 (0-0)**	0 (0-0)**	0 (0-0)**	0 (0-0)**
**Control**	3 (2-3)	3 (2-3)	4 (1-4)	9.5 (6-10)
**Dex.1**	0.5(0-1)**	0 (0-2)**	0 (0-2)**	1 (0-5)**
**RSP**	3 (1-3)	3 (1-3)	4 (1-4)	10 (4-10)
**AMT 5**	2.5 (1-3)	2.5 (1-3)	1.5 (0-4)	6.5 (2-10)
**AMT 10**	1 (1-1)**	1 (0-2)**	0(0-0)**	2 (1-3)**
**AMT 20**	0.5 (0-3)*	0.5 (0-3)*	0 (0-1)**	1 (0-7)**

Dex. 1=dexamethasone (1mg/kg), RSP= reserpine (6mg/kg), i.p. =intraperitoneally; Values are presented as median (range) of six rats in each group;

* P< 0.05,

** P<0.01 compared to control, Kruskal–Wallis followed by Mann-Whitney U test.

 In reserpine induced depressed rats which colitis were also induced, no significant differences between amitriptyline (10 mg/kg) and dexamethasone treated group were observed. Treatment with amitriptyline (10 mg/kg) and dexamethasone (1 mg/kg) were shown significant reduction in inflammation severity, inflammation extent and crypt damage (p<0.01). Total colitis, which is an overall indicator of the histological damage, was decreased in the amitriptyline (20 mg/kg)-treated group compared to the negative control group (P<0.01).

 The results in Table 3 also showed that there were no significant differences between amitriptyline at dose of 5 mg/kg and reserpine (RSP, 6 mg/kg) group in comparison to the negative control group.

*MPO Activity*

**Figure 8:**
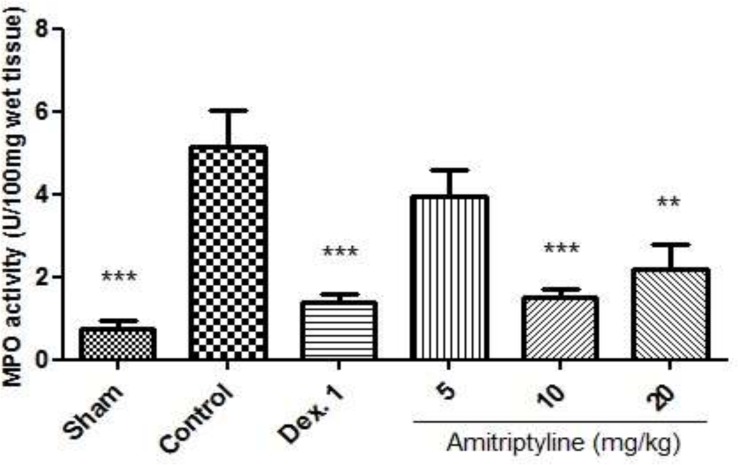
Effect of amitriptyline (5, 10, 20 mg/kg, i.p.) on myeloperoxidase (MPO) enzyme activity in the colonic tissue; i.p. =intraperitoneally, Dex.1=dexamethasone (1 mg/kg); Values are presented as mean ± S.E.M of six rats in each group; ** P<0.01, *** P<0.001 compared to control, one-way ANOVA followed by Tukey test

**Figure 9:**
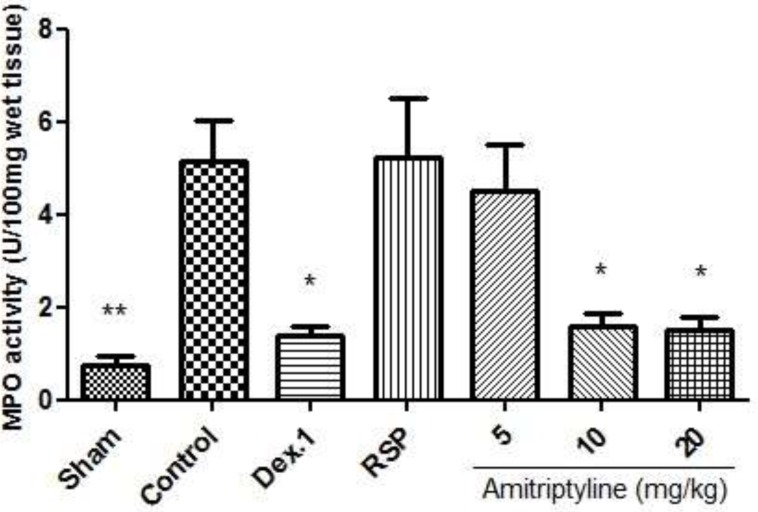
Effect of amitriptyline (5, 10, 20 mg/kg, i.p.) on myeloperoxidase (MPO) enzyme activity in the colonic tissue; Animals were reserpine induced (6 mg/kg) depressed and colitis was also induced; i.p. =intraperitoneally, RSP= reserpine, Dex.1=dexamethasone (1 mg/kg); Values are presented as mean ± S.E.M of six rats in each group; * P<0.05 compared to control, one-way ANOVA followed by Tukey test

 Colonic injury by acetic acid administration was accompanied by increased MPO activity, indicating neutrophil infiltration in inflamed tissue. This finding confirms the augmented leucocyte infiltration seen at histological inspection (Figure 8, 9).

As it is shown in Figure 8, MPO activity was diminished in treatment groups receiving amitriptyline (10 mg/kg) and dexamethasone (1 mg/kg) compared with control group (p<0.001). It is also decreased in amitriptyline at dose of 20 mg/kg significantly (p <0.01).

In reserpine induced depressed groups, we observed that MPO activity was conspicuously enhanced in the control and reserpine (6 mg/kg) groups. In addition, we found that amitriptyline (10, 20 mg/kg) and dexamethasone (1 mg/kg) significantly diminished the MPO activity level (P<0.05) (Figure 9).

## Discussion

It is clear that psychiatric illnesses are manifested in some patients with IBD, but it is much less clear that psychiatric illnesses play an etiological part in IBD. There are several investigations that show that psychiatric illnesses and depression in particular, co-occur with IBD more often than expected by chance (31). Although many types of antidepressant drugs, such as tricyclic antidepressants, SSRIs, MAOIs and many others have been known, but it is not yet a known choice to treat depression in people comorbid to UC. Rahimi *et al*. in a meta-analysis of randomized controlled trials in 2009 reported that TCAs exhibit clinically and statistically significant control of irritable bowel syndrome (IBS) symptoms and as IBS is very similar to IBD in pathogenesis but different in severity (32), it is worth to evaluate the effect of amitriptyline in this situation. So this research for the first time was conducted to follow that goal. We also evaluated that depression induction with reserpine (6 mg/kg) could exacerbate the experimental colitis and amitriptyline would be a good choice to treat depression in ulcerative colitis. 

Forced swimming test in rats is the most widely used method for assessing antidepressant activity in rat. Immobility time in this model is reduced by a variety of treatments which are therapeutically effective in depression (33, 34). Our findings clearly showed that amitriptyline at doses of 5, 10, 20 mg/kg (but not 2.5 mg/kg) has anti-depressive effect using forced swimming test. So these three doses of amitriptyline were selected to be evaluated in the ulcerative colitis rats.

Reserpine is a vesicular monoamines re-uptake blocker which depletes monoamines in the brain, and produces depression-like syndrome in animals. It is evaluated that reserpine at suitable doses can produce depression-like syndrome properly (35). Norepinephrine recovery following i.p. administration of reserpine happens from the fourth day and reaches normal levels at day 10 (36-38), so present study was designed on a five-days experiment in order to sure about the depressive effect of reserpine during the whole time treatment.

Another side of this research is UC and its probable relation with depression. As we know, induction of colitis in rats using acetic acid is a classical method used to produce an experimental model of human IBD. Several major causative factors in the initiation of human colitis such as enhanced vasopermeability, prolonged neutrophils infiltration, and increased production of inflammatory mediators are also involved in this animal model (2, 39).

To our knowledge, this is the first study which elucidates that i.p. injection of amitriptyline ameliorates acetic acid induced colitis in normal and reserpine induced depressed rats. It is clear that amitriptyline at doses of 10, 20 mg/kg improved macroscopic and histological scores of IBD and diminished the elevated amounts of biochemical markers such as MPO activity in normal and reserpine induced depressed rats.

Inhibition of catecholamine uptake by antidepressant drugs, making available more catecholamine at the receptor sites, initiates a number of reactions which ultimately lead to an inhibition of the inflammatory response. This might therefore be considered as a particular aspect of the potentiation of catecholamine response by antidepressant drugs. Indirect support for such an explanation is obtained from the results observed when the catecholamine stores have been depleted and/or noradrenaline biosynthesis has been inhibited (14, 20, 40). It is also clear that PMNs products play a key role in the amplification of inflammation and tissue damage in IBD (41). MPO which is located in the granules of neutrophils have been extremely useful as a marker of neutrophil infiltration to the site of inflammation (42). Nonspecific cellular immunity is also altered in IBD. Overproduction of monocytes and macrophages in both experimental and human IBD is largely implicated, probably because of an increased demand of macrophages in the inflamed gut (43- 45). Our results showed that, MPO activity elevated in both control and RSP groups and markedly attenuated by amitriptyline (10, 20 mg/kg) as reference drug. According to pathological examination, administration of amitriptyline elicited a marked reduction in the infiltration of leucocytes into the inflamed mucosa. 

There is also evidence of mast cell activation and degranulation in IBD. Enhanced histamine levels are common in the mucosa and intestinal secretions of patients with IBD, so can, but this is also likely to be a secondary, nonspecific event associated with inflammation (46). Considering the fact that amitriptyline, has anti-muscarinic and antihistamine effects, it is likely that ameliorative effect of amitriptyline in IBD may be partly due to anti-muscarinic and antihistamine effects.

The well-known theory of the anti-depressive effect of amitriptyline states that the drug modulates the synaptic concentrations of monoamine neurotransmitters such as norepinephrine and serotonin in the central nervous system (CNS). A number of *in-vivo* studies have shown that many antidepressants increase intracellular concentrations of cAMP through activation of monoamine receptors such as the receptors for serotonin and norepinephrine. It is demonstrated that TCA, SSRI and serotonin norepinephrine reuptake inhibitors (SNRI) inhibited IFN- gamma induced production of IL-6 and NO *in-vitro*, suggesting that the anti-inflammatory effects of various antidepressants are at least partially mediated by the cAMP dependent protein kinase A (PKA) pathway (47, 48). 

Hajhashemi and coworkers reported that i.p. and i.c.v. administration of amitriptyline has anti-inflammatory in carrageenan-induced paw edema in rats. They showed that amitriptyline elicited a marked reduction in the infiltration of PMN leucocytes into the inflamed tissue (19, 20). So i.p. administration of amitriptyline in the rat model of colitis also decreases the inflammation in the rat colonic mucosa through inhibition of PMN leucocytes infiltration. 

Sedatives produce analgesia and anti-inflammatory effect (49). Sedative effect of amitriptyline also has an important role to reduce inflammation and cause analgesia. It is worth mentioning that there is a strong connection between the inflammatory development and the generation of pain. Experimental studies have established that the inhibition of PMN cells migration and pro-inflammatory cytokines lessens the hyperalgesia evoked by different inflammatory stimuli. Therefore, it seems possible that the inhibitory effect of amitriptyline on leukocytes migration and TNF-alfa and IL-1 beta concentrations, at least partly, participates in its analgesic activity (48). 

In conclusion, it is tempting to speculate that antidepressants with anti-inflammatory effects such as amitriptyline could be useful treatments for ulcerative colitis in people including depression.
